# Supplementary material for: Seminoma and Embryonal Carcinoma Footprints Identified by Analysis of Integrated Genome-Wide Epigenetic and Expression Profiles of Germ Cell Cancer Cell Lines
Source: PLoS One. 2014 Jun 2;9(6):e98330. doi: 10.1371/journal.pone.0098330 (PMC4041891; doi:10.1371/journal.pone.0098330)
Supplement: File S1 — ZIP file containing DMRforPairs output for significant regions. Please start from the html files. (ZIP) [file pone.0098330.s008.zip › figures/16805.pdf]

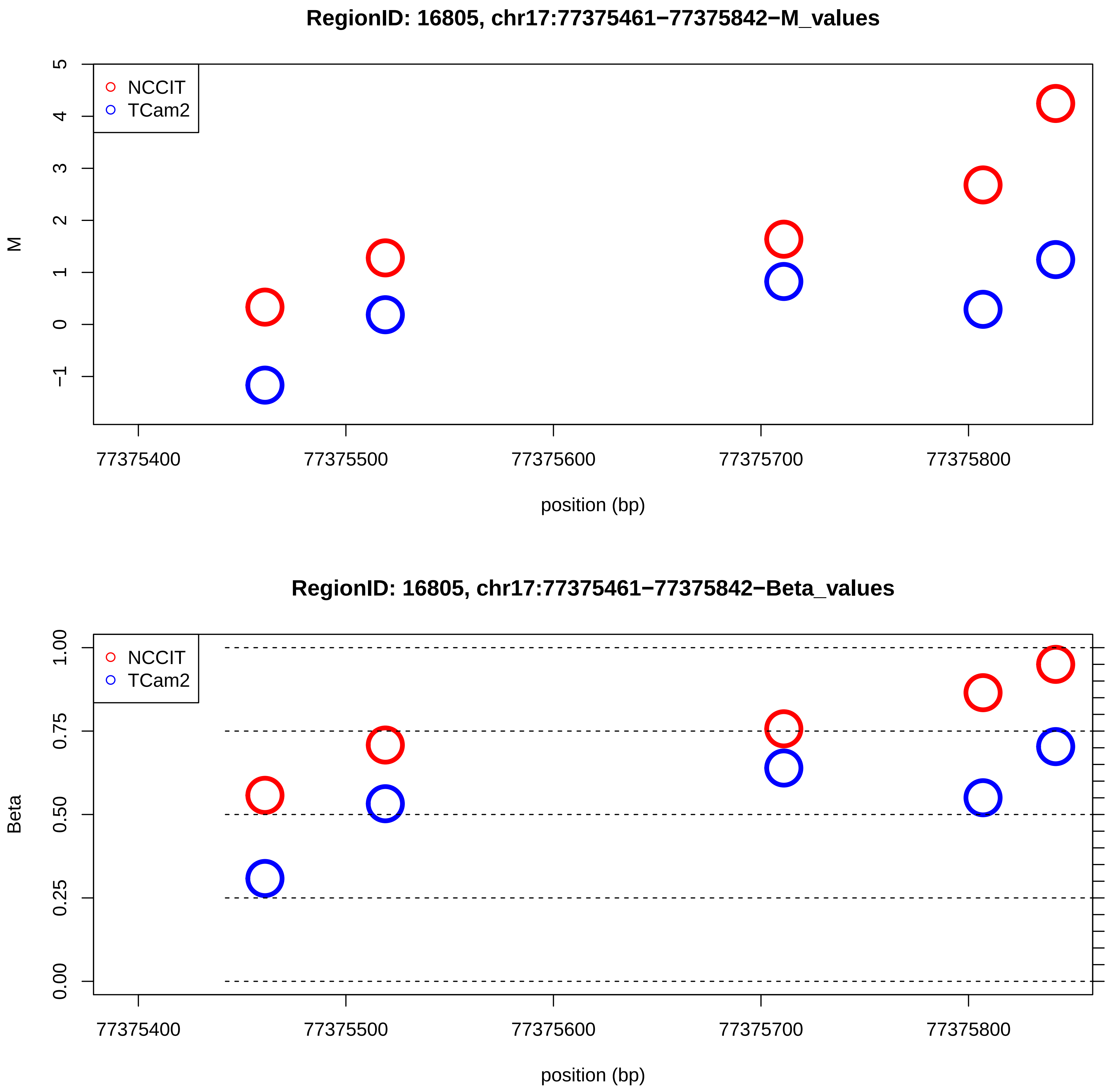
RegionID: 16805, chr17:77375461−77375842−M_values
NCCIT
TCam2
M
5
4
3
2
1
0
−1
77375400
77375500
77375600
77375700
77375800
position (bp)
RegionID: 16805, chr17:77375461−77375842−Beta_values
NCCIT
TCam2
Beta
1.00
0.75
0.50
0.25
0.00
77375400
77375500
77375600
77375700
77375800
position (bp)
